# Supplementary material for: Single Cell Transcriptomic and Chromatin Profiles Suggest Layer Vb Is the Only Layer With Shared Excitatory Cell Types in the Medial and Lateral Entorhinal Cortex
Source: Front Neural Circuits. 2022 Jan 26;15:806154. doi: 10.3389/fncir.2021.806154 (PMC8826650; doi:10.3389/fncir.2021.806154)
Supplement: Supplementary file 14 [file Data_Sheet_1.ZIP › LEC_Sup_vs_MEC_Sup_Motifs/homerResults/motif1.similar.html]

motif1

## Information for motif1

G
A
T
C
G
T
C
A
A
G
T
C
C
G
T
A
A
G
T
C
A
G
T
C
A
G
T
C
G
T
C
A
A
G
T
C
C
G
A
T
  
Reverse Opposite:  

G
C
T
A
A
C
T
G
A
C
G
T
C
T
A
G
A
C
T
G
A
C
T
G
G
C
A
T
A
C
T
G
C
A
G
T
C
T
A
G
  

|  |  |
| --- | --- |
| p-value: | 1e-167 |
| log p-value: | -3.865e+02 |
| Information Content per bp: | 1.729 |
| Number of Target Sequences with motif | 5679.0 |
| Percentage of Target Sequences with motif | 42.59% |
| Number of Background Sequences with motif | 6215.5 |
| Percentage of Background Sequences with motif | 31.19% |
| Average Position of motif in Targets | 283.4 +/- 221.8bp |
| Average Position of motif in Background | 327.7 +/- 249.5bp |
| Strand Bias (log2 ratio + to - strand density) | -0.0 |
| Multiplicity (# of sites on avg that occur together) | 1.30 |
| Motif File: | file (matrix) reverse opposite |

### Similar de novo motifs found

|  |  |  |  |  |  |  |  |
| --- | --- | --- | --- | --- | --- | --- | --- |
| Rank | Match Score | Redundant Motif | P-value | log P-value | % of Targets | % of Background | Motif file |
| 1 | 0.826 | G T A C G T A C G T C A A G T C T G A C G T A C G T C A A T G C G A T C G A T C G T C A A G T C | 1e-83 | -191.453313 | 36.22% | 28.47% | motif file (matrix) |
| 2 | 0.737 | T A G C C G T A A C T G C G T A A C T G C A T G T C A G A C T G A T C G G A T C | 1e-75 | -174.161973 | 25.92% | 19.36% | motif file (matrix) |
| 3 | 0.954 | A C T G C G A T C T A G A C T G A C T G G C A T A C T G C G A T | 1e-68 | -158.039002 | 21.40% | 15.63% | motif file (matrix) |
| 4 | 0.788 | G C T A G C A T G T A C G A T C G A T C G T A C G T A C C G T A T A G C C G T A C A G T G C T A | 1e-58 | -135.674360 | 13.08% | 8.83% | motif file (matrix) |
| 5 | 0.647 | A C T G A C T G A C T G A C T G A C T G A C T G A C T G A C T G | 1e-48 | -111.359050 | 74.99% | 69.23% | motif file (matrix) |
| 6 | 0.602 | C T A G C T A G T C A G C T G A C T A G C T A G C T A G C T A G T C A G T G A C C T A G C T A G | 1e-42 | -96.722161 | 29.54% | 24.35% | motif file (matrix) |
| 7 | 0.609 | G A C T T A G C G T A C T G A C C T G A A G T C G T A C A G C T G A T C G C A T | 1e-28 | -66.724550 | 15.38% | 12.09% | motif file (matrix) |
| 8 | 0.615 | A G T C G A T C G A T C C G A T A T G C G A T C G A T C G A T C G T A C G T A C C T A G A G C T | 1e-18 | -42.423763 | 11.80% | 9.48% | motif file (matrix) |
